# The Chemical Constituents and Anti-Complement Activity of Seven *Rhododendron* Species in Tibetan Medicine

**DOI:** 10.3390/molecules31132257

**Published:** 2026-06-26

**Authors:** Sujuan Wang, Yan Lu, Ke Zhang, Shiyan Wang, Shengnan Zhang, Hao Su, Ji De

**Affiliations:** 1 Key Laboratory of Biodiversity and Environment on the Qinghai-Tibetan Plateau, Ministry of Education, Xizang University, Lhasa 850000, China; wangsujuan71@stu.utibet.edu.cn (S.W.);; 2 School of Pharmacy, Fudan University, Shanghai 201203, China

**Keywords:** Tibetan medicine, *Rhododendron* species, UPLC-Q-TOF-MS, anti-complement activity, chemical fingerprinting

## Abstract

**Objective:** This study aims to explore the differences in chemical composition among Tibetan medicinal *Rhododendron* species and their potential correlation with anti-complement activity, with the goal of identifying promising medicinal resources. In Tibetan medicinal practice, the two groups of large-leaved *Rhododendron* (Tibetan: Dama) and small-leaved *Rhododendron* (Tibetan: Tali) are often used interchangeably despite unclear chemical and taxonomic bases. By comparing chemical profiles and evaluating anti-complement effects, this investigation seeks to provide preliminary scientific evidence for clarifying medicinal origins and facilitating the targeted development of high-quality resources. **Methods:** Ultra-performance liquid chromatography coupled with quadrupole time-of-flight mass spectrometry (UPLC-Q-TOF-MS) was employed to analyze seven *Rhododendron* samples. Separation was achieved on a Waters CORTECS UPLC C18 column (2.1 × 100 mm, 1.6 μm) using a gradient mobile phase system consisting of acetonitrile and 0.1% formic acid in water, at a flow rate of 0.3 mL/min and a column temperature of 30 °C. Data were acquired in both positive and negative electrospray ionization (ESI) modes. Compound identification was performed using Peakview 1.2 software by comparison with databases and literature. Grey relational analysis and partial least squares (PLS) regression, combined with 5000 bootstrap resampling iterations, were applied to establish spectrum–effect relationships and to screen for characteristic peaks potentially associated with anti-complement activity. **Results:** A total of 52 compounds were tentatively identified, including flavonoids (e.g., hyperin, isoquercitrin, taxifolin-3-O-arabinoside), terpenoids (e.g., grayanotoxin I/III), and chromanes (e.g., anthopogochromane series). The CH_50_ values of the ethanol extracts ranged from 179.29 to 579.47 μg/mL, with *Rhododendron principis* showing the strongest activity (179.29 ± 11.86 μg/mL), followed by *Rhododendron vellereum* (198.61 ± 7.93 μg/mL). Spectrum–effect analysis revealed that four unidentified peaks (F5315, F5822, F5368, F5991) exhibited negative regression coefficients and VIP means close to or above 0.8, suggesting a possible positive correlation with anti-complement activity. Among these, F5315 (VIP = 0.909), F5822 (VIP = 0.877), and F5368 (VIP = 0.834) showed relatively higher values and were considered preliminary candidate peaks warranting further investigation. **Conclusions:** This study tentatively identifies 52 compounds from the ethanol extracts of seven Tibetan medicinal *Rhododendron* species and reports their anti-complement activities. The findings reveal chemical distinctions between the large-leaved (Dama) and small-leaved (Tali) groups, offering a potential chemical basis for species differentiation and quality evaluation. Furthermore, four unknown peaks were preliminarily screened through spectrum–effect analysis as potential anti-complement candidates, which may serve as a foundation for future activity-guided isolation and quality marker studies.

## 1. Introduction

The genus *Rhododendron* L. is a highly diverse group within the family Ericaceae. There are approximately 962 known species worldwide [1], primarily distributed across Europe, North America, and Asia. East Asia and Southeast Asia are the two centers of diversification for the genus *Rhododendron* [2]. As one of the centers of diversity for this genus [3], the Himalayan region of China is home to numerous endemic species. According to the literature [4,5], there are more than 245 species belonging to 15 genera of the *Rhododendron* genus distributed in Tibet, ranging in elevation from 1000 to 5800 m. Among these, *Rhododendron mainlingense*, *Rhododendron anthopogon*, and *Rhododendron nivale* are primarily found in alpine regions at elevations of 2700–5800 m. These *Rhododendron* species are rich in flavonoids, terpenoids, and phenolic acids, and play an important role in Tibetan medicine.

According to *Chinese Tibetan Medicine*, in clinical practice, *Rhododendrons* are commonly classified into two categories based on leaf size: those with leaves longer than 4 cm are transliterated from Tibetan as “Dama,” such as the *Rhododendron principis* and the *Rhododendron vellereum*; Those with leaves smaller than 4 cm are collectively referred to as “Tali” (also known as “Balu”), and are further subdivided into “Tali Gabao” and “Tali Nabao.” Both the leaves and flowers of the Tali Gabao are used medicinally; representative species include the *Rhododendron anthopogon*, *Rhododendron mainlingense*, *Rhododendron lepidotum*, and the *Rhododendron fragariiflorum*. The flowers of Tali Nabao are used medicinally, with the representative species being *Rhododendron nivale* [6]. When used medicinally, it can alleviate cold-related ailments and treat diseases such as diphtheria and anthrax [7]. Dama is typically toxic and exhibits significant expectorant, antitussive, anti-inflammatory, and antibacterial effects. Although classical texts such as *Chinese Tibetan Medicine* do not fully document the medicinal uses of all species (such as *Rhododendron lepidotum* and *Rhododendron fragariiflorum*), these plants are indeed used in Tibetan medical practice. As important ornamental and medicinal plants, *Rhododendrons* have broad market prospects and tremendous development potential [8,9]. However, due to the wide variety of *Rhododendron* species and their high morphological similarity, there has long been confusion between the “Dama” and “Tali” groups during collection and processing, which has seriously compromised the consistency of herbal quality and the stability of clinical efficacy. Furthermore, existing research lacks a systematic comparison of the chemical compositions of “Dama” and “Tali”; the pharmacologically active components and taxonomic relationships of these species remain unclear, which hinders the scientific development and rational utilization of Tibetan *Rhododendron* resources.

In this study, ultra-performance liquid chromatography coupled with quadrupole time-of-flight mass spectrometry (UPLC-Q-TOF-MS) was employed to analyze the chemical constituents of the samples. This technique offers advantages such as high resolution, high sensitivity, and accurate mass measurement, making it suitable for the simultaneous detection and preliminary identification of multiple compound classes in *Rhododendron* species. Through systematic identification and comparison of the chemical components of “Dama” (large-leaved *Rhododendron*) and “Tali” (small-leaved *Rhododendron*), a chemical fingerprint was constructed to distinguish the misused species. Combined with the evaluation of anti-complement activity, the dominant medicinal resources were screened. This study aims to provide a theoretical basis and technical support for the original plant identification, quality evaluation, and resource development of Tibetan medicinal *Rhododendron*.

## 2. Results

### 2.1. Seven Phytochemicals in Rhododendrons

**Figure 1:**
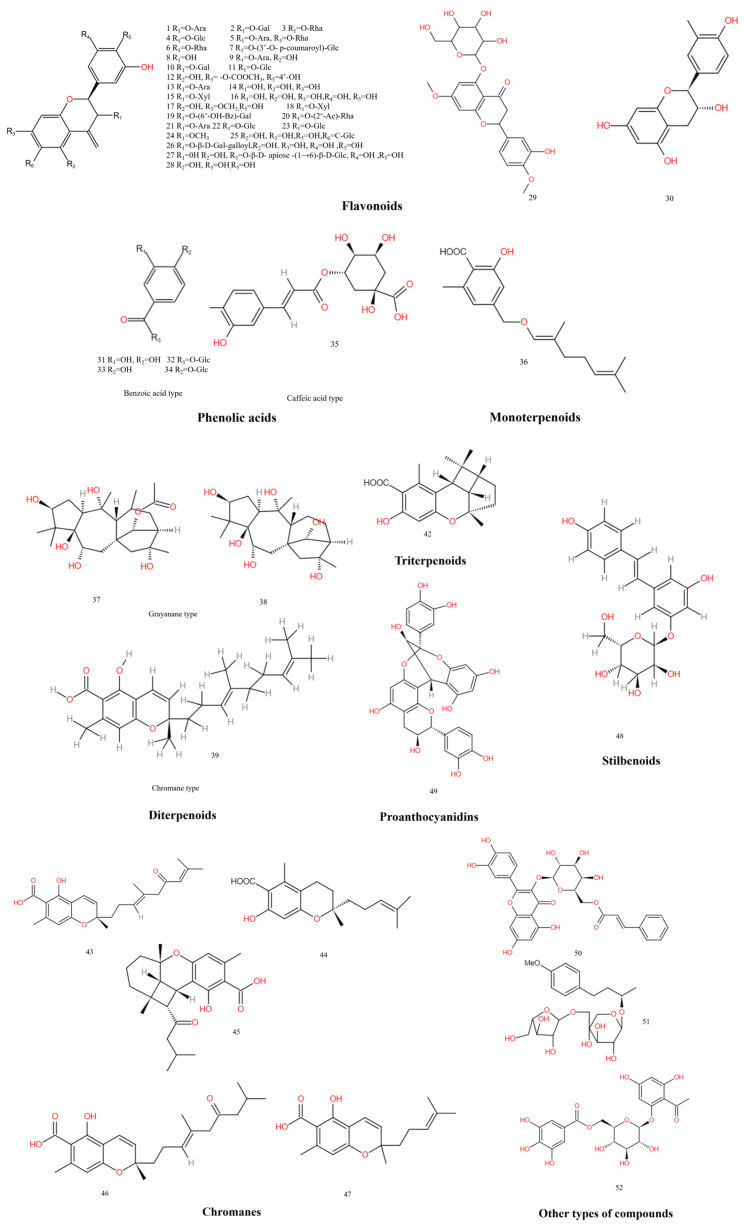
Structural formulas of 52 compounds from seven *Rhododendron* species.

Using the method described in Section 4.4, combined with a high-resolution mass spectrometry database of natural products and relevant literature, a total of 52 compounds were tentatively identified from seven *Rhododendron* species (chemical structures are shown in Figure 1; mass spectrometry data and compound classification are presented in Table S4). These compounds mainly include 30 flavonoids and their glycosides, comprising flavonol aglycones such as quercetin (8) and myricetin (16); flavonol glycosides such as hyperin (2) and quercitrin (6); flavone glycosides such as myricetin-3-O-arabinoside (21) and taxifolin derivatives (1, 18, 23); dihydroflavones such as farrerol (17); and flavanols such as catechin (30). In addition, five phenolic acids were tentatively identified, including protocatechuic acid (31) and chlorogenic acid (35); seven terpenoids, including ranhuadujuanine C (36), grayanotoxin I (38), nimbocinin (41), and anthopogocyclolic acid (42); five chromane/chromene derivatives (43–47); one proanthocyanidin, procyanidin A1 (49); one stilbenoid, resveratrol-3-O-glucoside (48); and three other compounds, including myrciaphenone B (52).

The number of compounds tentatively identified in each species and their representative constituents are as follows: 14 compounds were tentatively identified in *Rhododendron vellereum*, including protocatechuic acid (31), grayanotoxin I (38), and hyperin (2); 16 compounds in *Rhododendron principis*, including catechin (30), vanillic acid 1-O-β-D-glucoside (32), and rhododendrin (28); 15 compounds in *Rhododendron fragariiflorum*, including 4′-hydroxyacetophenone (33), myricetin (16), and farrerol (17); 14 compounds in *Rhododendron lepidotum*, including chlorogenic acid (35), catechin (30), and taxifolin-3-O-xyloside (18); 16 compounds in *Rhododendron mainlingense*, including taxifolin-3-O-arabinoside (1), quercitrin (6), and quercetin 3-(2″-acetylrhamnoside) (20); 16 compounds in *Rhododendron anthopogon*, including myricetin-3-O-β-D-galactoside (10), quercetin (8), and ranhuadujuanine C (36); and 16 compounds in *Rhododendron nivale*, including taxifolin-3-O-glucoside (23), persiconin (29), and farrerol (17).

### 2.2. A Comparison of the Phytochemical Composition of Seven Rhododendron Species

**Figure 2:**
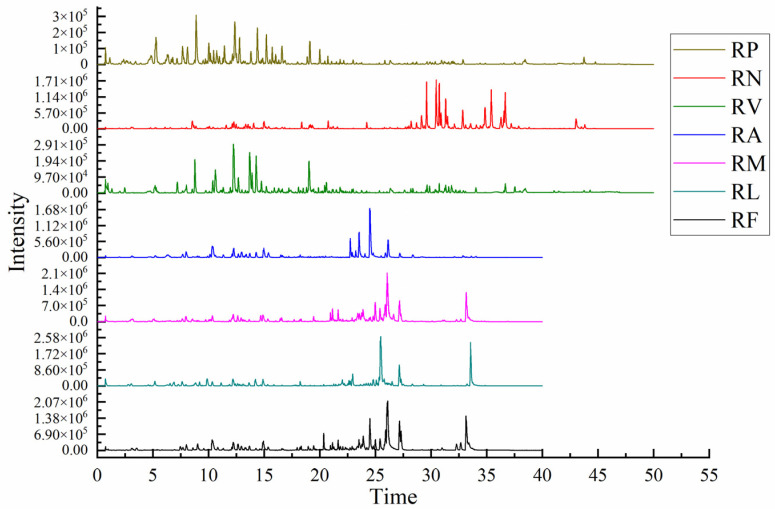
UPLC-HRMS base-peak ion flow diagram (BPC)-negative ion mode for seven species of *Rhododendron*. Caption: RP denotes *Rhododendron principis*, RN denotes *Rhododendron nivale*, RV denotes *Rhododendron vellereum*, RA denotes *Rhododendron anthopogon*, RM denotes *Rhododendron mainlingense*, RL denotes *Rhododendron lepidotum*, and RF denotes *Rhododendron fragariiflorum*. The same applies below.

**Figure 3:**
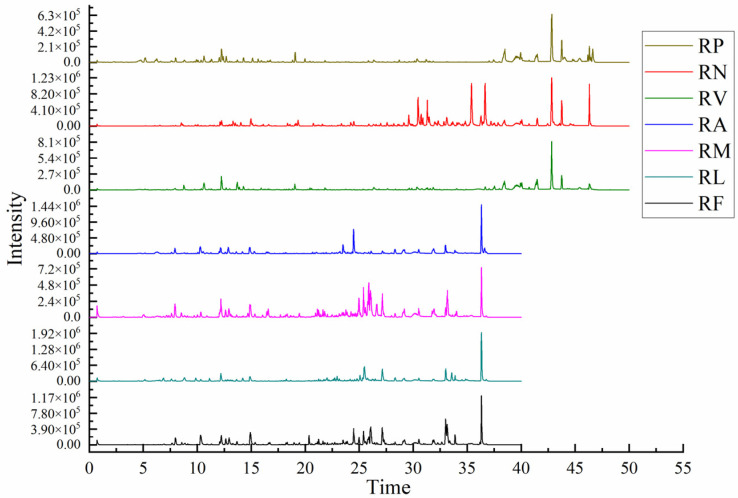
UPLC-HRMS base-peak ion flow diagram (BPC)-positive ion mode for seven species of *Rhododendron*.

**Figure 4:**
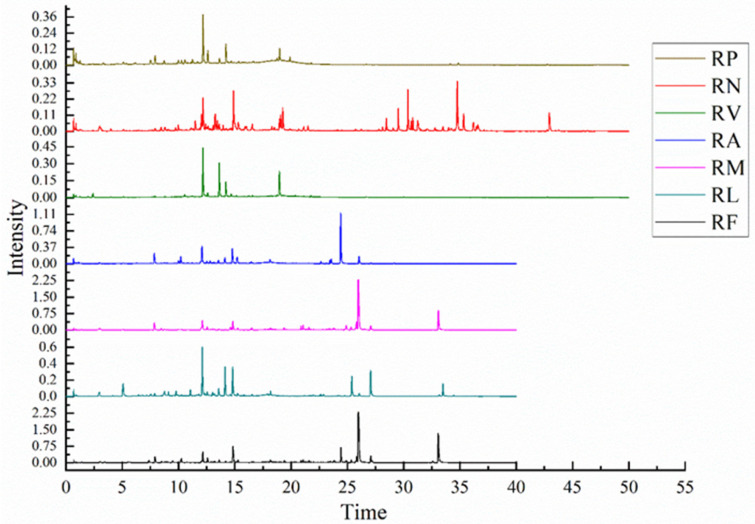
Seven species of *Rhododendron* UPLC chromatogram-UV 254 nm.

**Figure 5:**
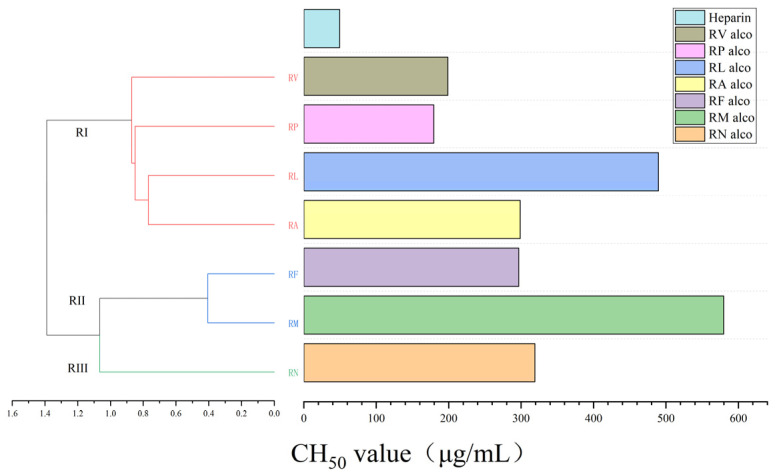
Clustering of seven *Rhododendron* chemical constituents with alcoholic extracts for anti-complement activity.

According to cluster analysis, the 52 tentatively identified compounds allowed the classification of the seven *Rhododendron* species into three chemotypes (see Figure 2, Figure 3, Figure 4 and Figure 5): RI (*Rhododendron vellereum*, *R. principis*, *R. lepidotum*, and *R. anthopogon*), RII (*R. fragariiflorum* and *R. mainlingense*), and RIII (*R. nivale*). The distribution of characteristic compounds in each group is as follows: In the RI group, the main detected constituents include taxifolin-3-O-arabinoside (1), procyanidin A1 (49), hyperin (2), isoquercitrin (3), avicularin (4), guaijaverin (5), etc. In the RII group, the main detected constituents include taxifolin-3-O-arabinoside (1), hyperin (2), isoquercitrin (3), myricetin-3-O-β-D-galactoside (10), myricetin-3-O-β-D-xylopyranoside (15), myricetin (16), rubiginosin B (40), anthopogochromene C (43), etc. In the RIII group, the main detected constituents include hyperin (2), quercetin (8), myricetin-3-O-glucoside (11), myricetin-3-O-β-D-xylopyranoside (15), myricetin (16), farrerol (17), anthopogocyclolic acid (42), daurichromenic acid (39), anthopogochromane (45), anthopogochromene A (46), laricitrin 3-O-β-D-glucoside (22), taxifolin-3-O-glucoside (23), persiconin (29), farrerol-7-O-β-D-apiofuranosyl(1 → 6)-β-D-glucopyranoside (27), myricetin 3-methyl ether (24), cirsimarin or isomer (25), etc.

Among the 52 tentatively identified compounds, the distribution of different constituents across the seven *Rhododendron* species exhibited marked differences. Hyperin (2) was a common component present in all seven species. Taxifolin-3-O-arabinoside (1) and isoquercitrin (3) were distributed in six species. Avicularin (4) and myricetin-3-O-β-D-xylopyranoside (15) were found in five species. Guaijaverin (5), myricetin-3-O-β-D-galactoside (10), myricetin (16), and anthopogochromenic acid (44) were distributed in four species. Procyanidin A1 (49), quercetin (8), anthopogocyclolic acid (42), nimbocinin (41), and daurichromenic acid (39) were found in three species. In addition, quercitrin (6), catechin (30), myricetin-3-O-glucoside (11), farrerol (17), rubiginosin B (40), anthopogochromene C (43), anthopogochromane (45), anthopogochromene A (46), laricitrin-3-O-β-D-glucoside (22), and other constituents were distributed in two species.

Among the 52 tentatively identified compounds, the distribution of different constituents across the seven *Rhododendron* species exhibited marked differences. Hyperin (2) was a common component present in all seven species. Taxifolin-3-O-arabinopyranoside (1) and isoquercitrin (3) were distributed in six species. Avicularin (4) and myricetin-3-O-β-D-xylopyranoside (15) were found in five species. Guaijaverin (5), myricetin-3-O-β-D-galactoside (10), myricetin (16), and anthopogochromenic acid (44) were distributed in four species. Procyanidin A1 (49), quercetin (8), anthopogocyclolic acid (42), nimbocinin (41), and daurichromenic acid (39) were found in three species. In addition, quercitrin (6), catechin (30), myricetin-3-O-glucoside (11), farrerol (17), rubiginosin B (40), anthopogochromene C (43), anthopogochromane (45), anthopogochromene A (46), laricitrin-3-O-β-D-glucoside (22), and other constituents were distributed in two species. Furthermore, each species possesses its own characteristic compounds: *Rhododendron vellereum* contains protocatechuic acid (31), grayanotoxin III (37), grayanotoxin I (38), and naringenin (9); R. principis contains vanillic acid-1-O-β-D-glucoside (32), rhododendrin (28), resveratrol-3-O-glucoside (48), phaeochrysin or isomer (51), rhododendrin methyl ether (12), azaleatin-3-O-arabinoside (13), and azaleatin (14); R. fragariiflorum contains 4′-hydroxyacetophenone (33); R. lepidotum contains vanillic acid-4-β-D-glucoside (34), chlorogenic acid (35), myrciaphenone B (52), taxifolin-3-O-xyloside (18), and 2″-galloylhyperin (26); R. mainlingense contains quercetin 3-(6″-p-hydroxybenzoylgalactoside) (19) and quercetin 3-(2″-acetylrhamnoside) (20); *R. anthopogon* contains myricetin-3-O-arabinoside (21), ranhuadujuanine C (36), and cannabiorcichromenic acid (47); R. nivale contains taxifolin-3-O-glucoside (23), persiconin (29), farrerol-7-O-β-D-apiofuranosyl(1 → 6)-β-D-glucopyranoside (27), myricetin 3-methyl ether (24), and cirsimarin or isomer (25).

### 2.3. Anti-Complement Activity Assay

The anti-complement activity of the ethanol extracts from seven *Rhododendron* species was evaluated in this study. The results showed that all samples exhibited varying degrees of complement inhibition, with CH_50_ values ranging from 179.29 to 579.47 μg/mL. Among them, the ethanol extract of *Rhododendron principis* showed the strongest activity, with a CH_50_ value of 179.29 ± 11.86 μg/mL, followed by that of *Rhododendron vellereum* (198.61 ± 7.93 μg/mL), while the extract of *Rhododendron mainlingense* exhibited the weakest activity (579.47 ± 11.49 μg/mL). The activities of the other four *Rhododendron* ethanol extracts fell between these values. The CH_50_ value of the positive control, heparin, was 49.25 ± 2.59 μg/mL, and all ethanol extracts showed weaker activity than heparin. The detailed results are presented in Table 1.

According to cluster analysis, the 52 tentatively identified compounds allowed the classification of the seven *Rhododendron* species into three chemotypes. RI (*Rhododendron vellereum*, *R. principis*, *R. lepidotum*, and *R. anthopogon*), RII (*R. fragariiflorum* and *R. mainlingense*), and RIII (*R. nivale*). This classification indicates significant differences in chemical composition among the different *Rhododendron* species. Species within the same chemotype may share similar metabolic characteristics, whereas compositional differences between chemotypes may be the intrinsic reason for their varying anti-complement activities (see Table 1 and Figure 5).

### 2.4. Results of the Spectrum–Effect Relationship Analysis of the Ethanol Extracts from Seven Rhododendron Species

#### 2.4.1. PLS Model Performance

A single-component partial least squares (PLS) regression model was established based on the eight selected characteristic peaks (F5991, F2582, F8140, F6758, F5822, F5315, F5368, F2481). The model exhibited a goodness-of-fit R^2^ of 0.908, a cross-validated predictive ability Q^2^ of 0.365, and a root mean square error (RMSE) of 0.303. The high R^2^ value indicates that the model can explain the variation in the training data reasonably well, whereas the low Q^2^ value (<0.5) suggests that, due to the extremely small sample size (*n* = 7), the predictive ability of the model is limited. Therefore, the results are primarily used as a preliminary screening reference for activity-related features.

#### 2.4.2. Overall Bootstrap Stability

**Figure 6:**
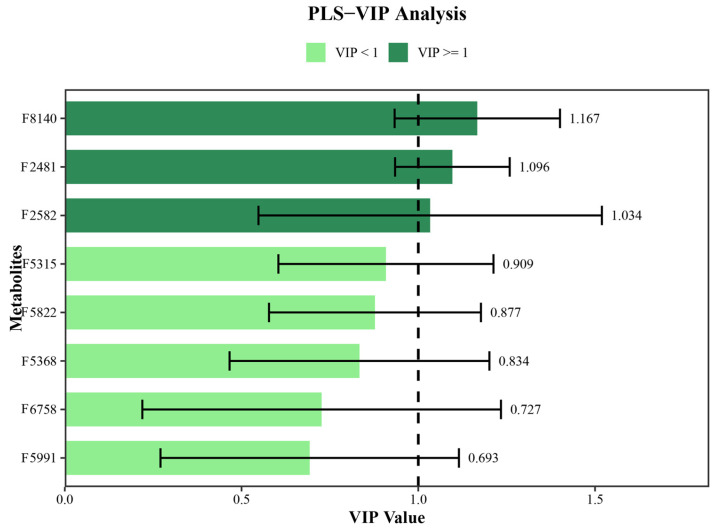
PLS-VIP Analysis Results of the Components of the Ethanol Extracts from Seven *Rhododendron* Species and Their Anti-complement Activity.

After 5000 bootstrap resampling iterations, the statistical results of the variable importance in projection (VIP) and regression coefficients for each feature are shown in Figure 6. Overall, the mean standard deviation of the VIP values was 0.348, and the mean standard deviation of the regression coefficients was 0.053, indicating high robustness of the parameter estimates for each feature. The VIP means of features F8140, F2481, and F2582 were 1.167, 1.096, and 1.034, respectively, all greater than 1.0, and their VIP_SD values were all less than 0.5, demonstrating high model contribution and stability.

#### 2.4.3. Regression Coefficient Analysis and Candidate Marker Screening

**Figure 7:**
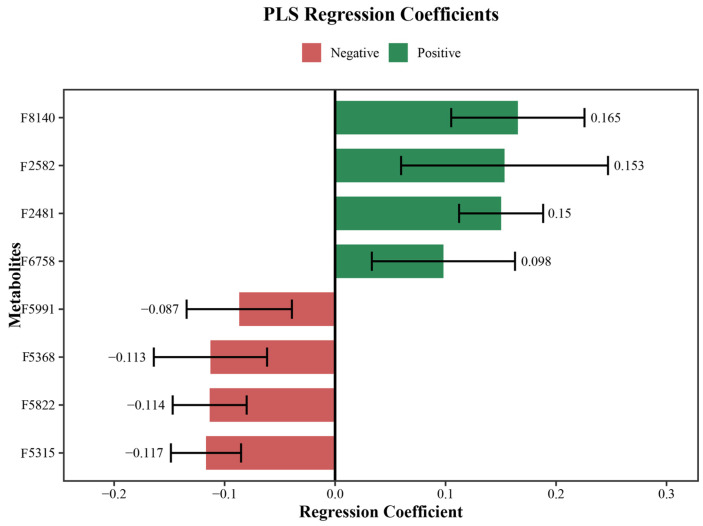
PLS Regression Coefficients of the Components of the Ethanol Extracts from Seven *Rhododendron* Species and Their Anti-complement Activity.

**Figure 8:**
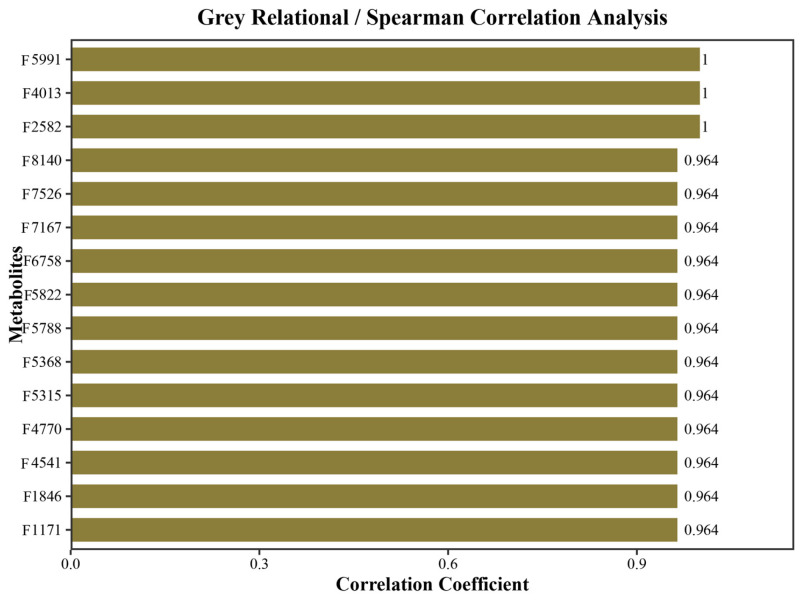
Spearman Correlation Analysis of the Components of the Ethanol Extracts from Seven *Rhododendron* Species and Their Anti-complement Activity.

Combined analysis of VIP and regression coefficients (Figure 7 and Figure 8) showed that although features F8140, F2481, and F2582 met the VIP criterion and exhibited good stability, they were excluded due to their positive regression coefficients (indicating a negative correlation between content and activity). Feature F6758 had a VIP_SD slightly above 0.5 (0.507), indicating relatively lower stability. According to the strict criteria (VIP_mean ≥ 1.0, VIP_SD < 0.5, Coefficient_mean < 0), no qualified quality marker was identified. Although their VIP means did not reach 1.0, features F5315, F5822, F5368, and F5991 satisfied Coefficient_mean < 0 and VIP_SD < 0.43, indicating a positive correlation between their content and anti-complement activity (higher content leads to lower CH_50_) and stable bootstrap results. These features were listed as potential active marker candidates.

Among them, features F5315 (VIP = 0.909, coefficient = −0.117), F5822 (VIP = 0.877, coefficient = −0.114), and F5368 (VIP = 0.834, coefficient = −0.113) had VIP means close to 0.9, warranting special attention in this small-sample study. Feature F5991 (VIP = 0.693, coefficient = −0.087), although contributing less, showed the correct direction and good stability, and can be considered as a secondary candidate.

## 3. Discussion

In this study, a total of 52 compounds were tentatively identified from seven Tibetan medicinal *Rhododendron* species using UPLC-Q-TOF-MS. Based on their structural features, these compounds can be classified into seven major categories, including flavonoids, terpenoids, and chromanes. This chemical classification reveals that flavonoids serve as the core constituents of *Rhododendron* species, while toxic diterpenes (e.g., grayanotoxins) and specific chromanes are also abundantly present [10], providing a chemical basis for subsequent quality marker discovery and bioactive compound screening.

The results of the chemical composition cluster analysis (RI–RIII) show a significant correlation with the traditional Tibetan medicinal classification of *Rhododendrons* (“Dama” and “Tali”). Specifically, the “Dama” group (*Rhododendron vellereum*, *Rhododendron principis*) occupies a similar position in the clustering. The flavonoids and phenolic acids abundant in this group are known to possess multiple pharmacological activities, including anti-inflammatory, analgesic, antioxidant, and cardioprotective effects. This aligns with Tibetan medical practices that utilize *Rhododendron principis* and *Rhododendron vellereum*, which are commonly used in Tibetan medical practice for expectorant, antitussive, and anti-inflammatory purposes. Similarly, the *Rhododendron fragariiflorum*, *Rhododendron mainlingense*, and *Rhododendron nivale* within the “Tali” group each form distinct clusters; their reported antihypertensive, hypoglycemic, and antioxidant activities provide modern scientific support for their traditional use in treating cold-related illnesses and diphtheria. The aforementioned chemical classification results provide a material basis for supporting the empirical classification of Tibetan medicines “Dama” and “Tali.”

This study correlates the tentatively identified constituents of *Rhododendron* species with the pharmacological activities reported in the literature, providing clues for elucidating the therapeutic potential of *Rhododendron* resources. For example, hyperoside exhibits various physiological activities, including anti-inflammatory, antispasmodic, diuretic, antitussive, antihypertensive, cholesterol-lowering, and central analgesic effects, as well as protection against cardiovascular and cerebrovascular diseases [11]. Phenolic acids such as protocatechuic acid exhibit significant cardioprotective effects, including anti-inflammatory, antioxidant, anti-atherosclerotic, and anti-myocardial ischemia properties [12,13]. As a flavonoid, naringin exhibits various pharmacological activities, including anti-inflammatory, antioxidant, antifibrotic, antitumor, and lipid metabolism-regulating effects [14,15]. The abundance of hyperoside, protocatechuic acid, and naringin in *R. vellereum* may contribute to the strongest anti-complement activity observed among the tested species.

The white rhodochrin and rhodochrin-B, which are unique to *Rhododendron principis*, have been shown to inhibit the activation of the TLR-7/NF-κB inflammatory pathway [16,17] and enzymes associated with neurodegenerative diseases, such as human glutamyl cyclase (hQC), and thus possess potential biological activity for the treatment of Alzheimer’s disease (AD) [18]. Phaeochrysin has been reported to be isolated and tentatively identified from *Rhododendron agglutinatum*, and this study also detected this compound in *Rhododendron principis* [19]. Furthermore, species-specific compounds have been identified in multiple species. For example, p-hydroxyacetophenone from *Rhododendron fragariiflorum* exhibits soothing and sebum-regulating effects [20], inhibits the in vitro adhesion, invasion, and migration of colon cancer cells, and reduces the metastatic burden in an in vivo model of colon cancer liver metastasis [21]. Chlorogenic acid (a phenolic compound) isolated from *Rhododendron lepidotum* exhibits potent inhibitory effects against various foodborne pathogens in vitro and in food matrices, as well as significant anti-inflammatory activity [22,23]; 2″-O-galloyl oleandrin demonstrates neuroprotective effects and some anti-inflammatory potential [24]; Myrciaphenone B (a phenethyl ketone derivative isolated from the Brazilian medicinal plant Myrciaria multiflora) was reported for the first time from this species [25]. Myrciarandol C (a monoterpene volatile compound) detected in *Rhododendron anthopogon* is a typical representative of naturally occurring volatile organic compounds in the carbon cycle of forest ecosystems [26]; Cannabiorcichromenic acid exhibits antibacterial activity [27]. Taxifolin-3-O-glucoside, detected in *Rhododendron nivale*, has hypoglycemic effects [28]; Cirsimarin, a flavonoid, can inhibit cell proliferation, migration, and invasion, and exhibits anti-inflammatory, antioxidant, lipolytic, and anti-obesity activities [29,30,31]. The discovery of these compounds provides a chemical basis for the diversified applications of these *Rhododendron* plants beyond their traditional uses in Tibetan medicine.

## 4. Materials and Methods

### 4.1. Equipment

Waters H-Class Ultra-High-Performance Liquid Chromatograph (Waters Technologies Co., Ltd., Shanghai, China), AB Sciex Triple TOF^®^ 4600 High-Resolution Mass Spectrometer (Sciex, Marlborough, MA, USA), Electronic Balance (ME104, Mettler-Toledo International Trading (Shanghai) Co., Ltd., Shanghai, China), Ultrasonic Cleaner (KQ-300 BD, Kunshan Ultrasonic Instrument Co., Ltd., Kunshan, China), High-Speed Centrifuge (SIGMA 3K15, SIGMA, Laborzentrifugen GmbH, Osterode am Harz, Germany), Constant-Temperature Shaker (WMZK 8002, Shanghai Medical Instrument Factory, Shanghai, China), Low-speed bench-top centrifuge (Model TDL-4, Shanghai Anting Scientific Instrument Factory, Shanghai, China), UV analyzer (ZF-20C dark box, Shanghai Jihui Scientific Analytical Instrument Co., Ltd., Shanghai, China), Analytical balance (Mettler-Toledo AG, Laboratory & Weighing Technologies, Greifensee, Switzerland), Rotary Evaporator (EYELA N-100, Tokyo Rika EYELA Co., Ltd., Tokyo, Japan), Low-Temperature High-Speed Centrifuge (HERAEUS FRESCO 17 Centrifuge, Waltham, MA, USA), Microplate reader (MULTISKAN MK3 model, Thermo Scientific, Waltham, MA, USA), Ultrasonic Bath (Model 4CQ50, Shanghai Ultrasonic Instrument Factory, Shanghai, China).

### 4.2. Reagents

Acetonitrile (MS grade, I0965929833, Merck, Darmstadt, Germany), methanol (mass spectrometry grade, I0931035804, Merck, Darmstadt, Germany), Water (Purified Water, Lot No.20210402C, Guangzhou Watsons Food & Beverage Co., Ltd., Guangzhou, China), Formic acid (MS grade, Lot No.Y9330090, CNW, Shanghai, China), Anti-SRBC antibodies (hemolysin) (in-house), Barbiturate buffer (5×, pH 7.4) (Beijing Leigen Biotechnology Co., Ltd., Beijing, China, Lot No.0314A17), Sterile sheep blood (SRB) (Lu Yaoying, Shanghai Reagent Supply Research Center, Shanghai, China), Heparin (Shanghai Aizite Biotechnology Co., Ltd., Shanghai, China, Lot No.090602).

### 4.3. Materials

#### Plant Material and Collection

From June to August 2019–2020, specimens of *Rhododendron nivale*, *Rhododendron principis*, *Rhododendron anthopogon*, *Rhododendron mainlingense*, *Rhododendron fragariiflorum*, *Rhododendron lepidotum*, and *Rhododendron vellereum* were collected in south-central Tibet. The plants used in this study were identified by the team led by Professor Laqiong from the School of Ecology and Environment at Tibet University. Detailed information on the sample collection is shown in Table S1 and Figure S1.

### 4.4. Experimental Methods

#### 4.4.1. UPLC-Q-TOF-MS

##### Preparation of the Test Solution

Approximately 0.5 g of the test sample was accurately weighed, placed in a stoppered conical flask, and mixed with 20 mL of 80% methanol. The mixture was subjected to ultrasonic treatment for 30 min (300 W, 40 kHz), then cooled to room temperature. The lost weight was replenished with 80% methanol, and the solution was shaken well. After sampling, the mixture was centrifuged at 12,000 rpm for 5 min, and the supernatant was collected for analysis.

##### UPLC-MS Chromatographic Conditions

Chromatography column: Waters CORTECS@ UPLC^®^ C18 (2.1 × 100 mm, 1.6 μm); Column temperature: 30 °C; Sample volume: 2 µL; Detection wavelength: 254 nm; Mobile phase composition and flow rate: Phase A is a 0.1% aqueous formic acid solution; Phase B is acetonitrile. See Table S2 for the gradient.

##### UPLC-MS Mass Spectrometry Conditions

Mass spectrometry detection mode: ESI-negative/positive ion mode. Mass spectrometry parameters: See Table S3.

To ensure the reliability and reproducibility of the analytical data, QC samples were inserted into the sample sequence, and a total of 10 injections were performed. The relative standard deviation (RSD) was calculated based on the peak areas of all analytes in the QC samples to assess the instrument’s stability. Analytes with an RSD ≤ 30% were considered stable and reliable. Seven *Rhododendron* samples were analyzed using ultra-high-performance liquid chromatography coupled with quadrupole-time-of-flight mass spectrometry (UPLC-Q-TOF-MS). Compounds were tentatively identified based on the multi-stage mass spectrometry data of the samples, in conjunction with a high-resolution mass spectrometry database of natural products and relevant literature. Data acquisition was performed using Analyst TF 1.7.1, and data processing was conducted using PeakView 1.2. During identification, mass spectrometry data were first matched against the Natural Products HR-MS/MS Spectral Library 1.0 database. Compounds were preliminarily screened based on the score information of each chromatographic peak, and further confirmed using the first- and second-order information of each peak. After normalization, fingerprint spectra were generated using Origin 2021.

### 4.5. Determination of Anti-Complement Activity in Seven Rhododendron Alcohol Extracts

#### 4.5.1. Sample Collection

The whole plants of the seven *Rhododendron* species were crushed. Approximately 100 g of each sample was weighed, then extracted with 95% ethanol under ultrasonic treatment for 1 h, followed by filtration. The residue was further extracted with purified water under reflux for 1 h, followed by filtration. The filtrates were concentrated and evaporated to dryness, and the obtained extracts were used for activity screening. The choice of 95% ethanol as the extraction solvent for the anti-complement activity assay was based on the following considerations: 95% ethanol enables a more comprehensive extraction of phenolic acids, flavonoids, triterpenes, and their glycosides from *Rhododendron* plants, which are potential anti-complement active substances; this solvent exhibited the best activity reproducibility and extraction efficiency in preliminary experiments; additionally, 95% ethanol is a widely used extraction solvent in natural product activity screening, facilitating cross-comparison with literature results.

#### 4.5.2. Preparation of the Solution

Prepare the following solutions: 1× barbitol buffer solution (BBS); 2% sheep erythrocyte suspension; 1:1000 hemolysin; test solutions (seven types of *Rhododendron* ethanol extracts); establishment of the classical complement hemolysis system: take complement (guinea pig serum), add 1× BBS, and dilute to prepare a solution, then determine the critical complement concentration [32].

#### 4.5.3. Classic Approach to Complement Inactivation Assay

Mix 0.2 mL of the critical complement concentration with 0.2 mL of the test sample, then add 0.1 mL of hemolysin and 0.1 mL of SRBC. Incubate in a 37 °C water bath for 30 min, then centrifuge in a low-temperature high-speed centrifuge at 5000 rpm and 4 °C. Transfer 0.2 mL of the supernatant from each tube to a 96-well plate and measure the absorbance at 405 nm using a microplate reader [33].

#### 4.5.4. Positive Control Complement Titer

In this experiment, heparin was selected as the positive control. Guinea pig serum was diluted with BBS to determine its complement titer.

#### 4.5.5. Component Cluster Analysis

In this experiment, cluster analysis of the seven *Rhododendron* samples was performed using Origin 2021 software. The presence or absence (binary variables) of the 52 chemical constituents in the seven samples was used as the clustering basis. The hierarchical clustering method (Ward‘s linkage method) was applied with Euclidean distance as the distance measure.

### 4.6. Spectrum–Effect Relationship Analysis of the Ethanol Extracts from Seven Rhododendron Species

#### 4.6.1. Data Preprocessing and Variable Screening

Based on the UPLC-Q-TOF-MS/MS raw data of the seven *Rhododendron* samples, a total of 8164 features (each tentatively identified by retention time RT and mass-to-charge ratio m/z) were obtained after processing with MS-DIAL software (ver.5.1.230912). After removing features with zero peak area in all samples, a feature matrix (7 samples × 8164 features) was generated. The anti-complement activity CH_50_ value (a smaller value indicates stronger activity) of the ethanol extract from each sample was used as the dependent variable Y.

Since the number of features far exceeded the number of samples, Spearman’s rank correlation coefficient was first used to perform a correlation analysis between each feature and CH_50_. The top 30 features (TOP_N = 30) with the highest absolute correlation coefficients were retained to reduce dimensionality. Subsequently, the Pearson correlation coefficients among the retained features were calculated, and variables with an absolute correlation coefficient greater than 0.95 (high collinearity) were removed. Finally, eight features (IDs: F5991, F2582, F8140, F6758, F5822, F5315, F5368, F2481) were obtained. Missing values were imputed with the median, and all features were Z-score standardized.

#### 4.6.2. Grey Relational Analysis

Grey relational analysis (GRA) was employed to investigate the correlation between each characteristic peak and the anti-complement activity. Before analysis, the peak areas of the common peaks were standardized, and the resolution coefficient (ρ) was set to 0.5. The grey relational grade (GRG) was calculated using the following formula:

$$r_{oi}=\frac{1}{m}\sum_{k=1}^{m}W_{k}\zeta_{i}(k)$$

where the standardized peak areas of the common peaks served as the comparison sequences, and the CH_50_ value of the ethanol extract from each sample served as the reference sequence. The calculations were performed using self-written code.

#### 4.6.3. Partial Least Squares Regression and Bootstrap Stability Analysis

Partial least squares regression (PLS) was used to establish a quantitative model between the chemical components (X) and the anti-complement activity (Y). Given the very small sample size (*n* = 7), to avoid overfitting, the number of PLS components was fixed to 1. To evaluate the stability of the model parameters, 5000 bootstrap resampling iterations were performed (with replacement, each sampling including 7 samples). In each iteration, the PLS model was refitted, and the variable importance in projection (VIP) and the standardized regression coefficient for each feature were calculated. Finally, the mean and standard deviation (SD) of the VIP and regression coefficient for each feature were used to represent its point estimate and stability. The predictive ability of the model was evaluated by the Q^2^ value from a 2-fold cross-validation.

#### 4.6.4. Screening Criteria for Quality Markers

A candidate quality marker had to simultaneously satisfy the following criteria: VIP_mean ≥ 1.0 (indicating a substantial contribution to the model); VIP_SD < 0.5 (indicating stable bootstrap results with low variation); and Coefficient_mean < 0 (since a smaller CH_50_ value corresponds to stronger activity, a negative coefficient indicates a positive correlation between content and activity). Features with a VIP_mean slightly below 1.0 but with a negative coefficient and good stability were also discussed as potential active component candidates.

## 5. Conclusions

In this study, UPLC-Q-TOF-MS/MS combined with PLS regression and bootstrap resampling was employed to preliminarily screen four characteristic peaks (F5315, F5822, F5368, and F5991) that were potentially associated with anti-complement activity. However, due to the limited sample size (*n* = 7), the predictive ability of the model (Q^2^ = 0.365) is suboptimal, and the screening results require further validation using an independent dataset with a larger sample size. In future studies, these candidate peaks will be subjected to targeted isolation and structural identification (e.g., preparative liquid chromatography, NMR, high-resolution mass spectrometry) to clarify their chemical identities, combined with in vitro activity assays to confirm their contribution to anti-complement activity. Such efforts would provide a more substantial scientific basis for elucidating the anti-complement active constituents and establishing quality markers for *Rhododendron* species. In addition, the present study is an untargeted metabolomics investigation. Although system stability was verified using QC samples, comprehensive method validation (including linearity, LOD, LOQ, and spike recovery) has not been fully performed. Targeted quantitative analyses should be carried out in the future to validate the key compounds.

In summary, this study represents a preliminary attempt to distinguish interspecific differences among Tibetan medicinal *Rhododendron* species using chemical fingerprinting combined with chemometric analysis, and to tentatively identify the metabolites of each species. By comparing with literature reports on known bioactive compounds, the medicinal potential of the seven *Rhododendron* species was preliminarily evaluated. Through correlation analysis with anti-complement activity, *Rhododendron vellereum* was highlighted as a promising resource with superior anti-complement activity. These findings provide a scientific basis and potential strategies for the botanical identification, quality evaluation, and innovative drug development of Tibetan medicinal *Rhododendron* plants. It should be noted that the extraction solvent used in this study was 95% ethanol, whereas traditional Tibetan medicine uses water decoctions. Therefore, the ethanol extract results may not fully reflect the actual pharmacologically active components. As all sample materials were consumed in the preliminary experiments, it was not possible to include water extract analyses in the current study. In future research, the sampling scope should be expanded, water extracts should be included for comparison, and the active components should be further verified through spectrum–effect relationship analysis.

## Figures and Tables

**Table 1:** CH_50_ values of anti-complementary activities of seven *Rhododendron* ethanol extracts ($\bar{x}$ ± SD, *n* = 3).

Sample	CH_50_ Value (μg/mL)
*Rhododendron fragariiflorum* alcohol extract (RF alco)	296.54 ± 18.39
*Rhododendron vellereum* alcohol extract (RV alco)	198.61 ± 7.93
*Rhododendron nivale* alcohol extract (RN alco)	318.97 ± 15.98
*Rhododendron lepidotum* alcohol extract (RL alco)	489.57 ± 17.91
*Rhododendron mainlingense* alcohol extract (RM alco)	579.47 ± 11.49
*Rhododendron anthopogon* alcohol extract (RA alco)	298.55 ± 6.96
*Rhododendron principis* alcohol extract (RP alco)	179.29 ± 11.86
Heparin	49.25 ± 2.59

## Data Availability

The original contributions presented in this study are included in the article. Further inquiries can be directed to the corresponding author.

## Supplementary Materials

The following supporting information can be downloaded at: https://www.mdpi.com/article/10.3390/molecules31132257/s1, Table S1: Longitude, latitude, altitude, and other relevant information of the *Rhododendron* sampling site; Figure S1: Sampling location of *Rhododendron*; Table S2: Mobile phase gradient; Table S3: Mass parameters (Sciex Triple TOF 4600 LC-MS); Table S4: Tentative Identification of the main components of seven species of *Rhododendrons* [34,35,36,37,38,39,40,41].
