# Supplementary material for: The Chemical Constituents and Anti-Complement Activity of Seven Rhododendron Species in Tibetan Medicine
Source: Molecules. 2026 Jun 26;31(13):2257. doi: 10.3390/molecules31132257 (PMC13362777; doi:10.3390/molecules31132257)
Supplement: Supplementary file 1 [file molecules-31-02257-s001.zip › molecules-4331352-supplementary.pdf]

Table S1 Longitude, latitude, altitude, and other relevant information of the *Rhododendron* sampling site

Number	Localities	Longitude and latitude	Elevation(m)	Plant
S1	Lebu Valley, Cuona County, Shannan Prefecture, Tibet	27°55′21″N 91°51′33″E	4244	*Rhododendron anthopogon*
S2	Naidula Pass, Yadong County, Shigatse, Tibet	27°23′49″N 88°50′4.92″E	4146	*Rhododendron principis*
S3	Lebu Valley, Cuona County, Shannan, Tibe	27°55′16″N 91°50′48″E	3966	*Rhododendron lepidotum*
S4	Bola Mountain, Cuona County, Shannan Prefecture, Tibet	27°57′23″N 91°53′33″E	4506	*Rhododendron fragariiflorum*
S5	Lulang Town, Bayi District, Nyingchi, Tibet	29°56′36″N 94°47′58″E	3700	*Rhododendron vellereum*
S6	Rido Township, Mozhugongka County, Lhasa, Tibet	29°42′58″N 92°2′2.76″E	4008	*Rhododendron mainlingense*
S7	Cuodo Township, Jiali County, Nagqu, Tibet	30°27′59″N 92°33′12.6E	4821	*Rhododendron nivale*

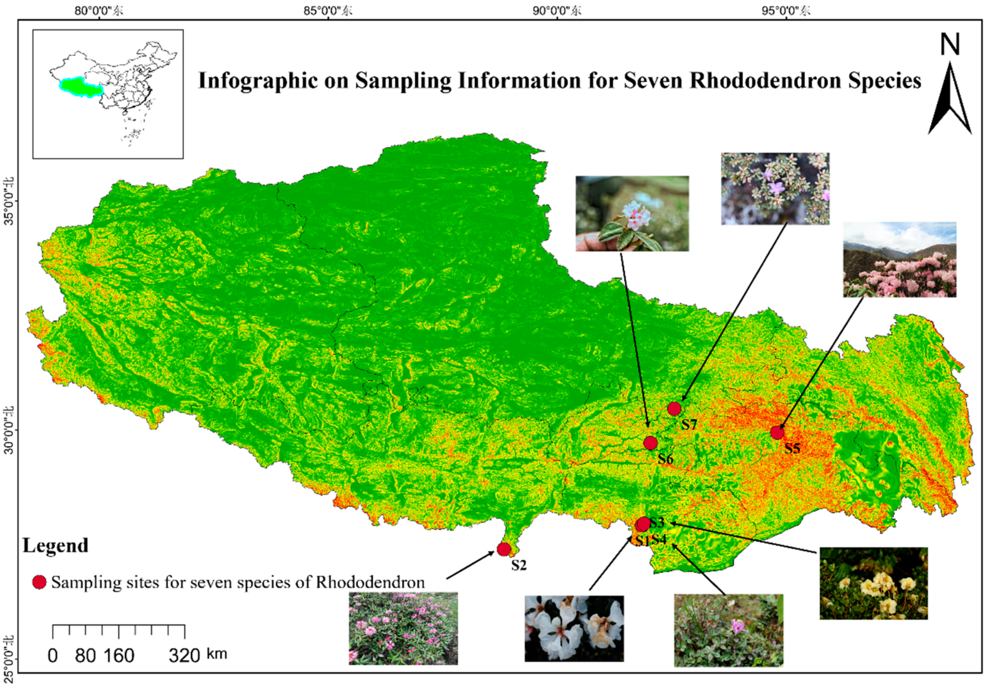

Figure S1 Sampling location of *Rhododendron*

Table S2 Mobile phase gradient

Time(min)	Flow Rate	B%
0	0.3	5
5	0.3	10
15	0.3	20
20	0.3	60
30	0.3	75
35	0.3	95
35.1	0.3	5
40	0.3	5

Table S3 Mass parameters (Sciex Triple TOF 4600 LC-MS)

MS parameter	Parameter value	MS/MS parameter	Parameter value
TOF mass range	50～1700	MS/MS mass range	50～1250
Ion Source Gas 1（psi）	50	Declustering Potential（V）	100
Ion Source Gas 2（psi）	50	Collision Energy（eV）	±40
Curtain Gas（psi）	35	Collision Energy Spread（eV）	20
Ion Spray Voltage Floating (V)	-4500/5000	Ion Release Delay（ms）	30
Ion Source Temperature (°C)	500	Ion Release Width（ms）	15
Declustering Potential（V）	100		
Collision Energy（eV）	10		

Table S4 Tentative Identification of the main components of seven species of *Rhododendrons*

No.	Time (min)	Observed adduct	Experimental m/z	Theoretical m/z	ppm	Molecular formula	Compound name	MS/MS data	Plant species	Classification	Reference
1	10.39	[M-H]-	435.097	435.0933	8.5	$C_{20}H_{20}O_{11}$	Taxifolin-3-O-arabinopyranoside	435.0934;285.0416;151.0048	A, B, C, D, E, F	Flavonoid glycoside	[35,39]
2	12.26	[M-H]-	463.0923	463.0882	8.9	$C_{21}H_{20}O_{12}$	Hyperin	463.0915;300.0293;271.0265;243.0277	A, B, C, D, E, F, G	Flavonol glycoside	[35,39]
3	12.69	[M-H]-	463.0927	463.0882	9.7	$C_{21}H_{20}O_{12}$	Isoquercitrin	463.0929;300.0303;271.0274;255.0328	A,B,C,D, E,F	Flavonol glycoside	[35,39]
4	13.71	[M-H]-	433.0817	433.0776	9.4	$C_{20}H_{18}O_{11}$	Avicularin	433.0827;300.0299;271.0268;255.0319;243.0318	A, B, C, E, D	Flavonoid glycoside	[35,39]
5	14.29	[M-H]-	433.0819	433.0776	9.8	$C_{20}H_{18}O_{11}$	Guaijaverin	433.0805;300.0293;271.0261;151.0048	A, B, E, D	Flavonoid glycoside	[35,39]
6	14.76	[M-H]-	447.0973	447.0933	9	$C_{21}H_{20}O_{11}$	Quercitrin	447.0950;301.0372;271.0250;255.0299;243.0320	A,F	Flavonol glycoside	[35,39]
7	18.83	[M-H]-	609.1301	609.125	8.4	$C_{30}H_{26}O_{14}$	Quercetin-3-O-(3'-O-p-coumaroyl)-glucoside	609.1304;463.0906;300.0290;271.0258;255.0314	A	Flavonoid glycoside	[39]
8	19.04	[M-H]-	301.0379	301.0354	8.4	$C_{15}H_{10}O_{7}$	Quercetin	301.0369;179.0000;151.0051;121.0306	A, D, G	Flavonol aglycone	[35,39]
9	20.6	[M+H]+	273.0782	273.0758	9	$C_{15}H_{12}O_{5}$	Naringetol	273.0790;153.0198;147.0453;119.0503;91.0552	A	Flavonol aglycone	[35,39]
10	8.112	[M-H]-	479.0859	479.0831	5.8	$C_{21}H_{20}O_{13}$	Myricetin-3-O-β-galactoside	479.0836;316.0221;287.0189;271.0243	B, C, F, D	Flavonoid glycoside	[35,39]
11	10.464	[M-H]-	479.0864	479.0831	6.9	$C_{21}H_{20}O_{13}$	Myricetin 3-O-β-D-Glucopyranoside	479.0851;316.0235;271.0256	B, G	Flavonoid glycoside	[35,39]
12	16.61	[M+FA-H]-	387.1668	387.1661	1.9	$C_{17}H_{26}O_{7}$	Rhododendrin methyl ether	387.1639;341.1599;161.0439;113.0249	B	Flavanone	[34,35]
13	19.11	[M-H]-	447.0961	447.0933	6.3	$C_{21}H_{20}O_{11}$	Azaleatin 3-O-arabinoside	447.0949;315.0519;300.0285;271.0255;255.0302;243.0306	B	Flavonol glycoside	[35,39,40]
14	20.002	[M-H]-	315.0531	315.051	6.6	$C_{16}H_{12}O_{7}$	Azaleatin	315.0534;300.0293;271.0267;255.0317;243.0317	B	Flavonol aglycone	[35,40]
15	14.94	[M-H]-	449.0744	449.0725	4.1	$C_{20}H_{18}O_{12}$	Myricetin-3-O-β-D-Xylopyranoside	449.0742;317.0304;178.9986;151.0039;137.0246	C, E, F, D, G	Flavonoid glycoside	[35,39]
16	15.36	[M-H]-	317.0316	317.0303	4.1	$C_{15}H_{10}O_{8}$	Myricetin	317.0300;271.0248;178.9991;151.0041;137.0248	C, F, D, G	Flavonol aglycone	[35,39]

No.	Time (min)	Observed adduct	Experimental m/z	Theoretical m/z	ppm	Molecular formula	Compound name	MS/MS data	Plant species	Classification	Reference
17	20.35	[M-H]-	299.0939	299.0925	4.7	$C_{17}H_{16}O_{5}$	Farrerol	299.0923;205.0504;193.0506;179.0350;135.0455;119.0605	C,G	Flavanone	[34,35,40]
18	9.879	[M+H]+	437.1076	437.1078	-0.5	$C_{20}H_{20}O_{11}$	Taxifolin 3-O-xylopyranoside	305.0658;287.0554;259.0606;231.0659;153.0185,149.0238,123.0443	E	Flavonoid glycoside	[35,39]

19	16.58	[M+H]+	585.1233	585.1239	-1	$C_{28}H_{24}O_{14}$	Quercetin-3-(6''-p-hydroxybenzoylgalactoside)	303.0503;283.0820;121.0280	F	Flavonoid glycoside	[39]
20	17.7	[M-H]-	489.1083	489.1039	9.1	$C_{23}H_{22}O_{12}$	Quercetin 3-(2''-acetyl-rhamnoside)	489.1093;447.0963;300.0297;271.0268;255.0318	F	Flavonoid glycoside	[39]
21	10.14	[M-H]-	449.0738	449.0725	2.8	$C_{20}H_{18}O_{12}$	Myricetin 3-Arabinoside	449.0749;316.0230;287.0193;271.0245;165.9907;139.0038	D	Flavonoid glycoside	[35,39]
22	12.13	[M-H]-	493.101	493.0988	4.5	$C_{22}H_{22}O_{13}$	Laricitrin 3-O-glucoside	493.1021;330.0393;315.0156;287.0205	D, G	Flavonoid glycoside	[35,39,40]
23	8.54	[M-H]-	465.1083	465.1039	9.6	$C_{21}H_{22}O_{12}$	Taxifolin-3-O-glucoside	465.1104;285.0437;151.0061;125.0261;107.0150	G	Flavonoid glycoside	[35,39]
24	19.165	[M-H]-	331.0486	331.0459	8	$C_{16}H_{12}O_{8}$	Annulatin	331.0500;316.0263;209.0119;181.0170;165.9932;139.0058;110.0028	G	Flavone derivatives	[35,39]
25	19.338	[M-H]-	475.129	475.1246	9.3	$C_{23}H_{24}O_{11}$	Cirsimarin or isomer	475.1287;312.0658;179.0360	G	Flavonoid glycoside	[35,39]
26	11.136	[M+H]+	617.1128	617.1138	-1.5	$C_{28}H_{24}O_{16}$	2''-galloylhyperin	315.0680,303.0482,153.0174	E	Flavonol glycoside	[39]
27	18.372	[M-H]-	593.1939	593.1876	10.7	$C_{28}H_{34}O_{14}$	Farrerol-7-O-β-D-apiofuranosyl(1→6)-β-D-glucopyranoside	593.1942;299.0944;179.0366;119.0516	G	Flavonol glycoside	[34,35]
28	8.894	[M-H]-	327.1467	327.1449	5.4	$C_{16}H_{24}O_{7}$	Rhododendrin	327.1453;165.0926;101.0250;59.0138	B	Flavonoids	[34,35]
29	14.054	[M-H]-	477.1449	477.1402	9.8	$C_{23}H_{26}O_{11}$	Persiconin	449.1530;315.0898;287.0949;271.0997;179.0356;153.0569	G	Flavonoid glycoside	[35,39]
30	5.279	[M+H]+	291.0869	291.0863	2	$C_{15}H_{14}O_{6}$	Catechin	207.0655;165.0549;147.0445;139.0393;123.0444	B, E	Flavanols	[35,39]
31	2.48	[M+H]+	155.0352	155.0339	8.5	$C_{7}H_{6}O_{4}$	Protocatechuic acid	137.0253;109.0283;93.0342;81.0336;65.0389	A	Phenolic acids	[35,39]
32	7.675	[M-H]-	329.0894	329.0878	4.8	$C_{14}H_{18}O_{9}$	1-O-vanilloyl-β-D-glucose	329.0872;209.0452;191.0350;167.0353;123.0454	B	Phenolic acids	[35,39]
33	7.47	[M-H]-	135.0463	135.0452	8.5	$C_{8}H_{8}O_{2}$	4'-Hydroxyacetophenone	135.0455;120.233;92.0271	C	Phenolic compounds	[35,39]
34	2.984	[M+H]+	331.102	331.1024	-1.1	$C_{14}H_{18}O_{9}$	Vanillic acid glucoside	193.0480,169.0499,151.0390,85.0276	E	Phenolic acids	[35,39]
35	5.155	[M+H]+	355.1022	355.1024	-0.4	$C_{16}H_{18}O_{9}$	Chlorogenic acid	163.0389,145.0284,135.0440	E	Phenolic acids	[35,39]

No.	Time (min)	Observed adduct	Experimental m/z	Theoretical m/z	ppm	Molecular formula	Compound name	MS/MS data	Plant species	Classification	Reference
36	22.74	[M-H]-	303.1616	303.1602	4.7	$C_{18}H_{24}O_{4}$	Ranhuadujuanine C	303.1596;259.1700;147.0452;135.0451;123.0451	D	Monoterpenoids	[34,35]
37	7.2	[M-+FA-H]	415.2366	415.2337	6.9	$C_{20}H_{34}O_{6}$	Grayanotoxin III	369.2303;351.2197;315.1978;297.1874;279.1761;149.0978	A	Diterpenoids	[34,35]
38	8.8	[M+FA-H]-	457.2478	457.2443	7.6	$C_{22}H_{36}O_{7}$	Grayanotoxin I	411.2410;369.2294;351.2176;333.2090;315.1965	A	Diterpenoids	[34,35]
39	33.16	[M-H]-	369.2093	369.2071	5.9	$C_{23}H_{30}O_{4}$	Daurichromenic acid	369.2093;351.1981;325.2185;187.0776;147.0461;135.0459;123.0457	C, F, G	Diterpenoids	[36,37,41]

40	25	[M-H]-	383.1864	383.1864	4.4	$C_{23}H_{28}O_5$	Rubiginosin B	383.1883;365.1771;339.1979;203.1451;189.0930;171.0823	C, F	Diterpenoids	[37,41]
41	27.17	[M-H]-	317.1777	317.1758	5.9	$C_{19}H_{26}O_4$	Nimbocinin	285.1505;241.1606;201.0560;185.0976;173.0979;159.0821;144.0587	C,F,E	Diterpenoids	[37,41]
42	24.51	[M-H]-	301.1145	301.1459	4.5	$C_{18}H_{22}O_4$	Anthopogocyclolic acid	301.1456;283.1349;257.1558;147.0452;135.0456;119.0507	C, D, G	Triterpenoids	[37,41]
43	25.42	[M-H]-	383.1883	383.1864	5	$C_{23}H_{28}O_5$	Anthopogochromene C	383.1887;365.1773;339.1979;191.0359;147.0460;135.0460	C, F	Chromanes	[37,41]
44	26.07	[M-H]-	301.1463	301.1445	5.9	$C_{18}H_{22}O_4$	Anthropochromenic acid	301.1459;283.1352;257.1556;187.0773;147.0460;135.0458;123.0459;79.0557	C, F, E, D	Chromanes	[37,41]
45	25.8	[M+H]+	387.2157	387.2166	-2.3	$C_{23}H_{30}O_5$	Anthopogochromane	369.2058;351.1943;285.1477;229.0855;205.0857;163.0386	F,G	Chromanes	[37,41]
46	26.63	[M+H]+	387.2162	387.2166	-1	$C_{23}H_{30}O_5$	Anthopogochromene A	369.2053;285.1477;229.0853;205.0857;177.0543;163.0387	F, G	Chromanes	[37,41]
47	23.53	[M-H]-	301.1448	301.1445	0.9	$C_{18}H_{22}O_4$	Cannabiorcichromenic acid	301.1445;283.1337;257.1543;188.0842;173.0611	D	Chromanes	[37,41]
48	11.43	[M-H]-	389.126	389.1242	4.6	$C_{20}H_{22}O_8$	Resveratrol 3-O-β-D-glucoside	227.0722;185.0619;143.0508	B	Stilbenoids	[35,39]
49	10.63	[M-H]-	575.1244	575.1195	8.5	$C_{30}H_{24}O_{12}$	Procyanidin A1	575.1218;539.1011;449.0896;407.0790;289.0722	A, B, D	Proanthocyanidins	[35,39]
50	21.84	[M-H]-	593.1351	593.1301	8.5	$C_{30}H_{26}O_{13}$	2-(3,4-Dihydroxyphenyl)-5,7-dihydroxy-3-[[6-O-(1-oxo-3-phenyl-2-propen-1-yl)-β-D-galactopyranosyl]oxy]-4H-1-benzopyran-4-one	593.1338;300.0286;271.0257;255.0305	A	Others	[39]
51	15.23	[M+FA-H]-	519.2113	519.2083	5.7	$C_{22}H_{34}O_{11}$	Phaeochrysin or isomer	473.2078;341.1625;161.0473;131.0362	B	Others	[35,39]
52	9.198	[M+H]+	483.1131	483.1133	-0.5	$C_{21}H_{22}O_{13}$	Myrciaphenone B	315.0708,297.0577,259.0587,193.0495.171.0277,153.0177	E	Others	[39]

Note: In the table, A refers to the *Rhododendron vellereum*, B to the *Rhododendron principis*, C to the *Rhododendron fragariiflorum*, D to the *Rhododendron anthopogon*, E to the *Rhododendron lepidotum*, F to the *Rhododendron mainlingense*, and G to the *Rhododendron nivale*. All compounds in the table were tentatively identified based on UPLC-Q-TOF-MS/MS spectral information and comparison with the high-resolution mass spectrometry database of natural products and literature, and have not been confirmed by reference standard
